# Usefulness of four surrogate indexes of insulin resistance in middle-aged population in Hefei, China

**DOI:** 10.1080/07853890.2022.2039956

**Published:** 2022-02-17

**Authors:** Rui Huang, Zi Cheng, Xingyi Jin, Xuemin Yu, Jinhui Yu, Yunpeng Guo, Li Zong, Jie Sheng, Xing Liu, Sufang Wang

**Affiliations:** a School of Public Health, Anhui Medical University, Hefei, China; b Hongguang Street Community Health Service Center, Hefei, China

**Keywords:** Insulin resistance, lipid parameters, odds ratio

## Abstract

**Objective:**

Previous study have shown that lipid accumulation product (LAP), visceral adiposity index (VAI), triglyceride/high-density lipoprotein cholesterol ratio (TG/HDL-C) and triglycerides/glucose index (TyG index) could be simple clinical indicators of insulin resistance (IR) based on anthropometric and/or biochemical parameters. However, the rational and preferred surrogate marker of IR in different population has yet to be validated. The aim of this study was evaluating the practicability of the LAP, VAI, TG/HDL-C, and TyG in predicting IR in middle-aged Chinese population.

**Methods:**

A cross-sectional study was conducted in 569 Chinese participants (mean age was 48.5; man 67.7%), and each participant completed a questionnaire survey, anthropometric measurement, and biochemical testing. One-way ANOVAs, Chi-squared test, Pearson’s correlation, and multiple logistic regression were used to evaluate the association between VAI, LAP, TG/HDL-C, and TyG with IR. To correctly discriminate individuals with insulin resistance, a receiver operating characteristic (ROC) analysis was conducted for each evaluated variable and the overall diagnostic accuracy was quantified using the area under the ROC curve (AUC). The AUC of evaluated variables were compared using a nonparametric approach. The optimal cut-off points were determined by the Youden’s index, and the corresponding sensitivity and specificity were provided.

**Results:**

Significant positive correlation was identified between HOMA-IR with TG/HDL-C (*r* = 0.306), VAI (*r* = 0.217), LAP (*r* = 0.381), and TyG (*r* = 0.371), respectively (all *p* < .001). After adjustment for potential confounders of IR, compared with the lowest tertiles, odds ratio (95% CI) having IR in the highest tertiles of TG/HDL-C, VAI, LAP and TyG were 6.07 (2.89–12.71), 10.89 (4.37–27.13), 4.68 (2.00–10.92), and 12.20 (5.04–29.56). The area under ROC curves to predict HOMA-diagnosed IR was 0.773 for TG/HDL-C, 0.767 for VAI, 0.806 for LAP, and 0.800 for TyG, respectively. Among those, LAP showed the greatest value of AUC [0.806 (0.763–0.850)] and highest specificity (0.804).

**Conclusion:**

Compared with other indicators, the LAP and TyG are simple, relatively accurate, clinically available surrogate markers of insulin resistance in middle-aged population in Hefei, China. Among 4 evaluated parameters, the LAP have the highest specificity and the TyG have the highest sensitivity.

**Key Messages**
- LAP and TyG could be used as simple and alternative methods to identify the individuals at risk for insulin resistance.
- LAP and TyG have relatively high predictive ability in diagnosis of IR compared with VAI and TG/HDL-C.
- No significant difference is observed between LAP and TyG in the ability of predicting insulin resistance.

## Introduction

Insulin resistance (IR) refers to the decline in the efficiency of insulin promoting glucose uptake and utilization for various reasons, and the body's compensatory secretion of excessive insulin produces hyperinsulinemia to maintain the stability of blood glucose [1]. It is widely known that IR is a major risk factor for type 2 diabetes mellitus (T2DM) and cardiovascular disease (CVD), and closely associated with other metabolic abnormalities [2–4]. These metabolic abnormalities contribute to the development of chronic non-communicable diseases worldwide.

On account of the clinical complexity, expensive equipment, and ethical reasons of hyperinsulinemic euglycemic glucose clamp (HEGC), which was originally developed by Defronzo [5] and regarded as the gold standard for measuring IR, homeostatic model assessment of insulin resistance (HOMA-IR) has been used as an alternative tool for defining IR [6]. However, fasting plasma glucose and fasting insulin were included for the calculation of HOMA-IR [6], and the measurement of insulin was limited due to its relative high cost in clinical practice. Consequently, actively searching for a simple, robust surrogate and cost-efficient biomarker to predict IR before the manifestation of clinical disease is of great importance.

Recently, several novel and easy-to-obtain tools for predicting IR were conceived. Triglyceride/high-density lipoprotein cholesterol ratio (TG/HDL-C), a simple marker deriving from two routine lipid parameters, has been shown to have the ability to predict IR and the risk of cardiovascular disease in non-diabetic individuals [7–9]. In addition, TG/HDL-C ratio could help clinicians to discriminate patients who are not only IR but also display the characteristic of dyslipidemia [10]. Visceral adiposity Index (VAI), a mathematical model based on anthropometric (body mass index (BMI), waist circumference (WC)) and biochemical parameters (TG and HDL-C), has been suggested to indirectly reflect visceral fat function [11]. Several researches have also reported that VAI could be used as a novel indicator of health-related outcomes such as cardiometabolic diseases and T2DM as well as IR [11–13]. Furthermore, it is well recognised that triglycerides/glucose index (TyG index), a product from the fasting levels of triglycerides and glucose, has high sensitivity for recognizing insulin resistance in apparently healthy subjects, compared with the HOMA-IR index [14]. TyG index has presented promising results as surrogate marker for the assessment of IR in some studies [14,15]. The superiority of the TyG index in identifying IR was also reported in many other studies [16,17]. Likewise, Lipid accumulation product (LAP), composed of WC and TG, was presented as a simple and effective tool for lipid over accumulation among adults by Kahn et al. [18]. LAP was designed for the U.S. National Health and Nutrition Examination Survey, and has shown better performance that of BMI for identifying higher total cholesterol, low-LDL-C, uric acid levels, higher total cholesterol/HDL-C and lower HDL-C levels among US adults [18]. More recently, several studies have suggested that LAP can used as a capable marker of metabolic syndrome, IR and diabetes risks [18–20].

In previous studies, these screening tools had ever used as clinical marker of IR alone [7,12,14,19]. Nevertheless, there could be difference in the ability to predict IR in different ethnic populations. To date, data for direct comparison of the four indicators to predict HOMA-diagnosed IR in different population by age and sex are limited. Hence, we aimed to determine a relatively superior marker associated with IR in a middle-aged population in Hefei, China.

The purposes of this study are as follows: (1) To assess the ability of TG/HDL-C, VAI, TyG, and LAP for strength and independence as predictive signs for IR; (2) To evaluate the diagnostic accuracy of TG/HDL-C, VAI, TyG, and LAP in identifying IR in different population by age and sex, and then compared.

## Subjects and methods

### Study population

We conducted a cross-sectional study from November 2019 to January 2020 in Hefei city, Anhui Province, China. A total of 743 subjects aged 40–60 years who work in Anhui Hong Sifang Co. Ltd, China Salt Group (CNSG), were invited to the Hongguang Community Health Service Centre for a physical examination. Participants were interviewed face to face by trained investigators using structured questionnaires which included information on educational attainment, cigarette smoking and drinking status, histories of current and previous illness, and medical treatment. Inclusion criteria were long-term residents of local districts aged ≥40 years, no serious mental illness and participants volunteered to participate in the survey. A total of 174 individuals were excluded because of incomplete information about their physical examination or laboratory assessments. Finally, 569 subjects were enrolled in the final analysis.

Hong Sifang Cohort was established in CNSG Anhui Hong Sifang Co., Ltd. which is a large scale state owned chemical industry. We aimed to explore the relationship between exposure to environmental pollutants, life-style factors, and chronic cardiometabolic diseases. Data of this study came from baseline survey of Hong Sifang Cohort. All participants volunteered for the study, and all the respondents signed informed consent. This study was reviewed by the Ethics Committee of Anhui Medical University.

### Anthropometric

Height and weight were measured twice, and the average value was taken when the subjects were barefoot and wearing light indoor clothing. Body mass index (BMI) was calculated by dividing body weight (in kilograms) by the square of height (in meters). At the end of the exhalation, waist circumstance (WC) was measured with a non-elastic tape at the midpoint between the bottom of the chest cavity and the top of the iliac crest. The trained nurse measured blood pressure twice with a mercury sphygmomanometer. Normally, the measurement was carried out in a sitting position after the subjects rested for at least 10 min. Thereafter, the mean of the two measurements was determined as the blood pressure of the subject. Body composition was measured with Inbody570 body composition analyzer (Inbody.co.Ltd, Seoul, Korea).

### Biochemical measurements

The night before the blood was collected, participants were asked to fast for at least 12 h. 20 ml fasting venous blood were collected by nurses in the physical examination centre, and centrifugation was completed in 2 h. We used serum for biochemical measurements, and all measurements were completed in 24 h. Fasting blood glucose, triglycerides, total cholesterol, low-density lipoprotein cholesterol, high-density lipoprotein cholesterol and other biochemical indicators were detected with Hitachi 7180 automatic biochemical analyzer (Hitachi, Tokyo, Japan). Fasting insulin concentration was measured using the radioimmunology assay (Gamma counter XH-6020, Hefei, China).

### Definitions

Smoking is defined as at least one cigarette a day for more than 6 months, and drinking was defined as at least three times a week for more than six months [21]. Homeostatic model assessment of insulin resistance was calculated using the following formula: HOMA-IR = insulin (mU/L)×fasting glucose (mmol/L)/22.5 [22]. IR was defined as HOMA-IR >2.69, based on an epidemiology survey conducted in China [23]. Referring to the physical activity assessment methods in the United States, the physical activity levels for each participant were measured as metabolic equivalent in hours per week (MET-h/week) in which different intensities of physical activities were assigned to corresponding MET levels [24].

The following insulin resistance indexes were calculated according to the published formula: TG/HDL-C as the ratio of serum triglycerides and high-density lipoprotein cholesterol [7]; VAI as [WC (cm)/39.68 + (1.88 × BMI)] × [TG (mmol/L)/1.03] × [1.31/HDL-C (mmol/L)] for men and [WC (cm)/36.58 + (1.89 × BMI)] × [(TG (mmol)/0.81] × [1.52/HDL-C (mmol/L)] for women [11]; LAP as [(WC (cm) − 65)×TG (mmol/L)] for men and [(WC (cm) − 58) × TG (mmol/L)] for women [18]. And TyG index was calculated as Ln [fasting triglycerides (mg/dL) × fasting glucose (mg/dL)/2] [14].

Central obesity was defined as according to waist circumference ≥90 cm for men and waist circumference ≥80 cm for women, according to the recommendations of the International Diabetes Federation for Asians [25]. Based on the recommended standards for Chinese adults, BMI (kg/m^2^) was divided into 4 categories: underweight: 18.5; normal weight: 18.5−23.9; overweight: 24.0−27.9; obesity: 28 and above [26]. Hypertension was defined as the level of systolic blood pressure ≥140 mmHg or/and diastolic blood pressure level ≥90 mmHg or being medically treated now [27].

### Statistical analysis

Continuous variables were described by means and standard deviations (SD), or medians and interquartile ranges for variables that do not follow normal distribution through Kolmogorov-Smirov test. Categorical variables were described by frequency. Differences in general demographic and clinical characteristics between IR group and non-IR group were compared using student’s t-test or Mann-Whitney U test, respectively, and the chi-square test was used for categorical variables. Non-normally distributed variables were log-transformed (natural logarithm) to approximate normal distributions before conducting the linear regression analyses.

Multivariate logistic regression analyses were performed to assess the risk of insulin resistance, the four indicators entered the regression model according to their tertiles, respectively. The odds ratios (OR) and the corresponding 95% confidence intervals (95% CI)were calculated. A receiver operating characteristic (ROC) curve was used to evaluate the ability of TG/HDL-C, VAI, LAP and TyG to correctly distinguish IR. The diagnostic accuracy on predicting prevalent insulin resistance was examined through area under the receiver operating characteristic curve (AUC) with 95% CIs. Differences between the AUC of evaluated variables were performed with a nonparametric approach (DeLong test) [28]. The drawing of ROC curve and comparisons of AUCs were achieved according to MedCalc program. The optimal cut-off points of sex- and age-specific were determined by the Youden’s index (YI) [29], which was calculated according to the corresponding sensitivity, specificity for each marker.

All statistical analyses were performed by using SPSS software (version 24.0, applicable to windows SPSS, Chicago, Illinois, USA). The significance level was set at *p* < .05.

## Results

Among the 569 participants, the average age was 48.3 years and 67.7% were men, and 115 (20.2%) were diagnosed as insulin resistance. Table 1 showed the general demographic and clinical characteristics of the study population related to insulin resistance. The levels of SBP, DBP, waist circumference, waist-to-hip ratio, BMI, and body fat content of IR group were significantly higher than non-IR group (all *p<* .001). In terms of biochemical indicators, significantly higher fasting insulin, fasting plasma glucose, TC, TG, LDL-C, TG/HDL-C, VAI, LAP and TyG, and lower HDL-C were observed in IR group compared with non-IR group (all *p* < .001). There were no significant differences between IR and non-IR group in financial income, smoking status, drinking status and educational attainment and physical activity.

**Table 1.** Characteristics of the participants according to IR.

Variables	Non-insulin resistance	Insulin resistance	*P* Value
N (%)	454 (79.8)	115 (20.2)	–
Age (years)	48.3 ± 4.7	49.2 ± 5.2	.067
Male [*n* (%)]	300 (66.1)	85 (73.9)	.066
Educational attainment (%)			
Middle or less than middle	99 (21.8)	28 (24.3)	.591
High school	297 (65.4)	76 (66.1)	
College or above college	58 (12.8)	11 (9.6)	
Per capita income, [CNY/year, %]			
≤10,000	49 (10.8)	13 (11.3)	.516
10,000–30,000	85 (18.7)	25 (21.7)	
30,000–60,000	210 (46.3)	54 (47.0)	
≥60,000	110 (24.2)	23 (20.0)	
Current smoker [*n* (%)]	157 (34.6)	37 (32.2)	.496
Current drinker [*n* (%)]	102 (22.5)	26 (22.1)	.466
Physical activity (MET-h/week)	102.9 (11.2–20.1)	108.5 (11.0–19.7)	.751
WC (cm)	85.6 ± 7.8	94.0 ± 8.4	<.001
WHR	0.88 ± 0.06	0.94 ± 0.06	<.001
BMI (kg/m^2^)	23.54 ± 2.58	26.63 ± 3.15	<.001
SBP (mmHg)	132.9 ± 16.9	143.7 ± 17.6	<.001
DBP (mmHg)	82.7 ± 10.8	89.2 ± 9.8	<.001
Body fat mass (kg)	16.8 (13.6–20.7)	22.1 (18.4–25.4)	<.001
Fat %	25.2 (21.7–29.5)	29.7 (25.8–32.9)	<.001
FPG (mmol/L)	5.00 (4.69–5.31)	5.65 (5.21–6.70)	<.001
Fasting insulin (mIU/ml)	45.23 ± 17.38	110.25 ± 71.88	<.001
HOMA-IR	1.39 (1.01–1.81)	3.43 (2.91–4.28)	<.001
TG (mmol/L)	1.17 (0.83–1.69)	2.09 (1.46–3.24)	<.001
TC (mmol/L)	4.84 ± 0.86	5.22 ± 1.12	<.001
HDL-C (mmol/L)	1.38 (1.18–1.62)	1.18 (1.05–1.29)	<.001
LDL-C (mmol/L)	2.67 (2.25–3.13)	2.81 (2.43–3.37)	<.001
TG/HDL-C ratio	0.86 (0.56–1.32)	1.88 (1.18–2.89)	<.001
TyG	8.46 (8.12–8.86)	9.17 (8.76–9.74)	<.001
VAI	2.22 ± 2.76	4.60 ± 4.09	<.001
LAP	27.45 (17.41–43.86)	61.89 (36.90–107.58)	<.001
History of diabetes [*n* (%)]	52 (11.5)	15 (13.0)	.370
Medication used^a^, [*n* (%)]	32 (7.0)	15 (13.0)	<.001

Note: Data are expressed as mean ± standard deviation or median (interquartile range) and frequency (percentage) as appropriate; *P* values are for the Student’s t-test, Mann–Whitney test and χ2 analyses across the groups.

CNY: China Yuan (1CNY = 0.155 USD); WC: waist circumference; WHR: waist-to-hip ratio; BMI: body mass index; SBP: systolic blood pressure; DBP: diastolic blood pressure; FPG: fasting plasma glucose; HOMA-IR: homeostasis model assessment of insulin resistance; TG: triglycerides; TC: total cholesterol; HDL-C: high-density lipoprotein cholesterol; LDL-C: low-density lipoprotein cholesterol; TyG: triglycerides/glucose index; VAI: visceral adiposity index; LAP: lipid accumulation product; TG/HDL-C: triglyceride-to-HDL cholesterol ratio, respectively.

^a^Indicating any self-reported medication used in the past 2 weeks.

The results of correlation analyses indicated that TG/HDL-C, VAI, LAP and TyG were all significantly correlated with fasting insulin, fasting plasma glucose, and HOMA-IR index (all *p* < .001). After further adjusting for age and sex, we found that this correlation still existed (all *p* < .001). As shown in Table 2, LAP was the most strongly correlated with fasting insulin (*β* = 0.390, *p* < .001) and HOMA-IR (*β* = 0.385, *p <* .001), whereas TyG was the most strongly correlated with FPG (*β* = 0.480, *p* < .001).

**Table 2.** Pearson's correlation and multiple linear regression models of evaluated variables associated with metabolism indexes.

	FINS (pmol/l)			FPG (mmol/L)			HOMA-IR index		
	*r*	*β*	*P* Value	*r*	*β*	*p* Value	*r*	*β*	*P* Value
Ln TG/HDL-C	0.303	0.317	<.001	0.264	0.234	<.001	0.306	0.311	<.001
Ln TyG	0.312	0.327	<.001	0.494	0.480	<.001	0.371	0.383	<.001
VAI	0.202	0.209	<.001	0.210	0.174	<.001	0.217	0.216	<.001
Ln LAP	0.380	0.390	<.001	0.299	0.274	<.001	0.381	0.385	<.001

Note: *r*: correlation coefficient; *β*: Standardised regression coefficient; Multiple regression analysis is adjusted for age and sex.

TyG: triglycerides/glucose index; VAI: visceral adiposity index; LAP: lipid accumulation product; TG/HDL-C: triglyceride/HDL cholesterol ratio; FPG: fasting plasma glucose; FINS: fasting insulin; HOMA-IR: homeostasis model assessment of insulin resistance.

**Figure 1.**
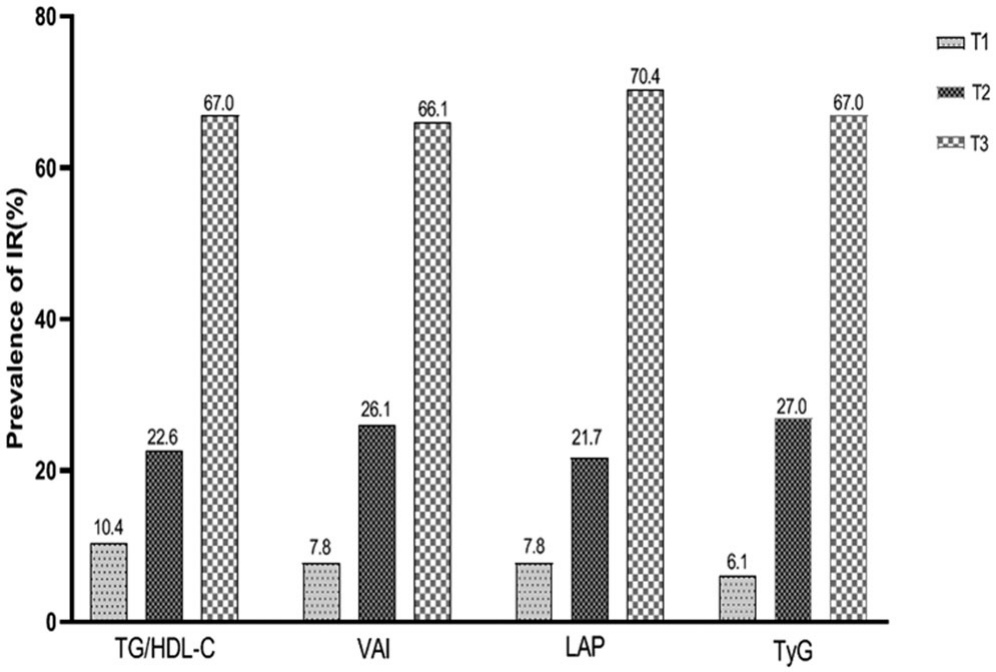
The prevalence of IR by tertiles of TG/HDL-C, VAI, LAP and TyG.

We further studied the prevalence of insulin resistance in the tertiles of four evaluated variables. As shown in Figure 1, the prevalence of insulin resistance increased greatly with the elevated tertiles of TG/HDL-C, VAI, LAP and TyG (all *p* < .001). The comparative ORs and 95% CI in the tertiles of each variable were presented in Table 3. Among them, in the three models, TyG showed the strongest correlation with insulin resistance, when the highest tertile was compared with the lowest tertile across all logistic regression models. Model 1 was unadjusted and Model 2 adjusted for age, sex and BMI for TG/HDL-c and TyG, and only age and BMI for VAI and LAP. Despite adjusting for a number of possible confounders (including age, sex, BMI, WC, SBP, current smoker, current drinker, educational attainment, physical activity level, per capita income, medication treatment and history) had weakened the association (model 3), there was still statistically significance for 4 evaluated variables (all *p* < .001), when the highest tertile was compared with the lowest tertile. Subgroup analyses were conducted to explore the increased risk of insulin resistance with each tertile increase of evaluated parameters in different subgroups (Table 4). Results showed that there were still correlation between four evaluated parameters and the increased risk of insulin resistance across all stratified factors. (all *p* < .001).

**Table 3.** The risk of IR according to tertiles of evaluated variables.

Variables	ORs (95% CI)					
	Model 1	*P* Value	Model 2	*P* Value	Model 3	*P* Value
TG/HDL-C						
T1	1.00 (reference)	–	1.00 (reference)	–	1.00 (reference)	–
T2	2.35 (1.15–4.81)	.019	1.56 (0.72–3.39)	.263	1.57 (0.72–3.46)	.261
T3	10.19 (5.31–19.58)	<.001	6.15 (2.98–12.65)	<.001	6.08 (2.90–12.73)	<.001
P value for trend		<.001		<.001		<.001
TyG						
T1	1.00 (reference)	–	1.00 (reference)	–	1.00 (reference)	–
T2	5.09 (2.18–11.89)	<.001	4.20 (1.71–10.29)	.002	4.06 (1.64–10.04)	.002
T3	17.97 (8.01–40.35)	<.001	13.04 (5.46–31.11)	<.001	12.20 (5.04–29.56)	<.001
P value for trend		<.001		<.001		<.001
VAI						
T1	1.00 (reference)	–	1.00 (reference)	–	1.00 (reference)	–
T2	3.77 (1.74–8.18)	.001	2.48 (1.09–5.61)	.029	2.17 (0.93–5.05)	.073
T3	13.53 (6.52–28.06)	<.001	6.67 (3.08–14.41)	<.001	6.80 (2.95–15.68)	<.001
P value for trend		<.001		<.001		<.001
LAP						
T1	1.00 (reference)	–	1.00 (reference)	–	1.00 (reference)	–
T2	3.05 (1.38–6.72)	.006	1.56 (0.68–3.59)	.293	1.49 (0.63–3.54)	.357
T3	15.08 (7.28–31.25)	<.001	5.37 (2.43–11.84)	<.001	4.84 (2.08–11.27)	<.001
P value for trend		<.001		<.001		<.001

Note: Model 1 unadjusted. Model 2 adjusted for age and BMI for VAI and LAP, added sex for TG/HDL-C and TyG. Model 3 adjusted for age, BMI, WC, SBP, current smoker, current drinker, educational attainment, physical activity level, per capita income and history of diabetes for VAI and LAP, added sex further for TG/HDL-C and TyG.

OR: odds ratios; CI: confidence interval. TyG: triglycerides/glucose index; TG/HDL-C: triglyceride-to-HDL cholesterol ratio; VAI: visceral adiposity index; LAP: lipid accumulation product.

**Table 4.** The risk of IR for 1-tertile increase of evaluated variables by sex, age, BMI.

	ORs (95% CI)							
	TG/HDL-C	*P* Value	VAI	*P* Value	LAP	*P* Value	TyG	*P* Value
Insulin resistance								
Sex^a^								
Men	2.74 (1.89–3.95)	<.001	3.28 (2.17–4.95)	<.001	2.44 (1.63–3.67)	<.001	3.34 (2.27–4.92)	<.001
Women	4.29 (1.89–9.73)	<.001	5.12 (2.19–11.95)	<.001	3.25 (1.39–7.58)	<.001	7.80 (2.88–21.11)	<.001
Age^b^								
<48 years	2.62 (1.51–4.54)	<.001	3.71 (1.93–7.13)	<.001	2.16 (1.17–2.96)	<.001	3.66 (1.99–6.73)	<.001
≥48 years	3.72 (2.08–6.67)	<.001	3.66 (1.99–6.72)	<.001	3.19 (1.73–5.89)	<.001	4.08 (2.24–7.44)	<.001
BMI^c^								
Normal	2.30 (1.35–3.93)	<.001	1.97 (1.02–3.82)	<.001	2.87 (1.63–5.04)	<.001	2.46 (1.29–4.67)	<.001
Overweight or obesity	4.02 (2.38–6.79)	<.001	5.34 (2.91–9.81)	<.001	3.71 (2.04–6.74)	<.001	4.56 (2.64–7.86)	<.001

Note: Data are ORs with 95% CI. a: adjusted for age, BMI, WC, SBP, current smoker, current drinker, educational attainment, physical activity level, per capita income and history of diabetes; b: adjusted for sex, BMI, WC, SBP, current smoker, current drinker, educational attainment, physical activity level, per capita income and history of diabetes; c adjusted for age, sex, WC, SBP, current smoker, current drinker, educational attainment, physical activity level, per capita income and history of diabetes.

All variables were calculated for 1-tertile increasing of evaluated variables.

BMI: body mass index; TG/HDL-C: triglyceride-to-HDL cholesterol ratio; TyG: triglycerides/glucose index; VAI: visceral adiposity index; LAP: lipid accumulation product, OR: odds ratios; CI: confidence interval, respectively.

**Figure 2–6.**
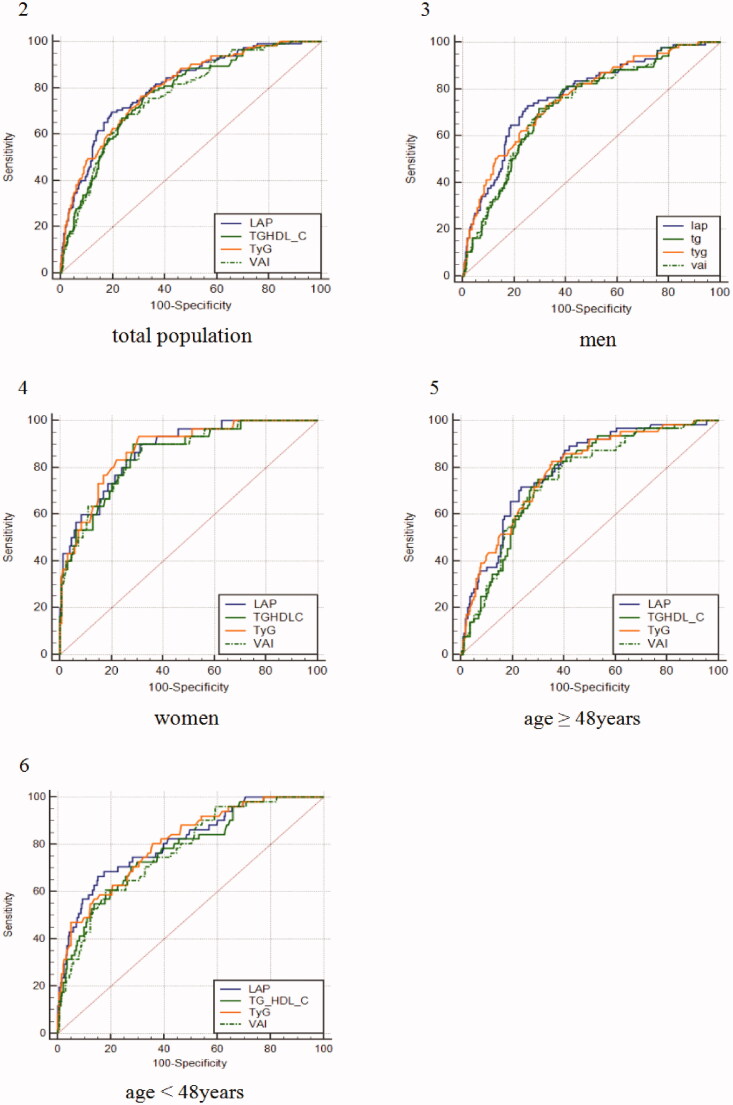
Comparison of the diagnostic value of triglycerides/glucoseindex (TyG index), triglyceride-HDLcholesterol (TG/HDL-C), lipid accumulation product (LAP) and visceral adiposity index (VAI) by age and sex. (2) total population; (3) men; (4) women; (5) age ≥48 years; (6) age <48 years.

ROC analysis of four evaluated indicators was performed. As shown in Table 5, concerning the ability to predict IR, LAP had the highest AUC (0.806 [0.763–0.850]), followed by TyG, TG/HDL-C and VAI, with the AUCs ranging from 0.800 to 0.767 in the total study population. The sensitivity (0.765) of TyG was highest. On the contrary, LAP had the highest specificity (0.804). Analyses stratified by age and sex (Figure 2–6) found that LAP and TyG showed higher diagnostic values than TG/HDL-C and VAI (*p* < .05 for difference), whereas LAP showed no difference compared with TyG between sex and age. The best diagnostic performance of TyG was found in women when compared with TG/HDL-C (0.877 [0.821–0.921] vs. 0.855 [0.795–0.902], *p* = .045) and VAI (0.877 [0.821–0.921] vs. 0.855 [0.796–0.902], *p* = .048). Meanwhile, there is no statistical significance compared with LAP. Furthermore, results were observed that LAP showed the optimal predictive power for IR both in study participants younger than 48 years (AUC: 0.811 [0.761–0.854], *p* < .05), older than 48 years (AUC: 0.794 [0.741–0.840], *p* < .001), and men (AUC: 0.773 [0.728–0.814], *p* < .05).

**Table 5.** Comparison of predictive accuracy and cut-off values of TG/HDL-C, VAI, LAP, and TyG by age and sex for the total sample.

	AUC (95% CI)	*p* Value	Sensitivity	Specificity	Cut-off	Youden Index
Overall						
LAP	0.806 (0.763–0.850)^b-c^	–	0.696	0.804	48.70	0.500
TyG	0.800 (0.757–0.844)^b-c^	<.628	0.765	0.692	8.76	0.457
TG/HDL-C	0.773 (0.737–0.807)^a^	<.001	0.765	0.678	2.68	0.446
VAI	0.767 (0.721–0.813)^a^	<.001	0.661	0.764	2.41	0.427
Age <48						
LAP	0.811 (0.761–0.854)^b-c^	–	0.667	0.846	48.70	0.513
TyG	0.807 (0.765–0.845)^b-c^	.760	0.804	0.643	8.62	0.447
TG/HDL-C	0.775 (0.722–0.821)^a^	.043	0.706	0.722	2.71	0.455
VAI	0.771 (0.718–0.818)^a^	.047	0.608	0.817	2.54	0.450
Age ≥48						
LAP	0.794 (0.741–0.840)^b-c^	–	0.719	0.765	49.64	0.484
TyG	0.785 (0.732–0.832)^b-c^	.549	0.828	0.648	8.75	0.476
TG/HDL-C	0.760 (0.705–0.806)^a^	.015	0.750	0.700	2.87	0.449
VAI	0.752 (0.697–0.802)^a^	.010	0.828	0.601	2.08	0.429
Men						
LAP	0.773 (0.728–0.814)^b-c^	–	0.729	0.747	48.70	0.476
TyG	0.763 (0.718–0.805)^b-c^	.494	0.729	0.677	8.87	0.406
TG/HDL-C	0.732 (0.685–0.775^)a^	.004	0.718	0.703	3.11	0.421
VAI	0.735 (0.688–0.778)^a^	.004	0.729	0.673	2.54	0.403
Women						
LAP	0.873 (0.816–0.917)	–	0.900	0.688	29.25	0.588
TyG	0.877 (0.821–0.921)^b-c^	.854	0.933	0.695	8.50	0.628
TG/HDL-C	0.855 (0.795–0.902)	.334	0.900	0.714	2.00	0.614
VAI	0.855 (0.796–0.902)	.131	0.900	0.682	1.08	0.582

Note: a indicates a significant difference compared with TyG; b indicates a significant difference compared with TG/HDL-C; c indicates a significant difference compared with VAI.

*P* values are calculated by the nonparametric approach to compare AUC of other evaluated variables with LAP.

TG/HDL-C: triglyceride-to-HDL cholesterol ratio; TyG: triglycerides/glucose index; VAI: visceral adiposity index; LAP: lipid accumulation product; AUC: area under the receiver operating characteristic curve, respectively.

## Discussion

By exploring the usefulness of TG/HDL-C, VAI, LAP and TyG as predictors of HOMA-diagnosed IR in middle-aged Chinese population, we found that LAP and TyG had better performance in predicting IR at sample-specific optimal cut-off than TG/HDL-C and VAI, based on the highest point estimated of AUC and YI. In spite of lower predicting capability of LAP and TyG for IR, they seemed to have better availability in identifying IR, whereas TG/HDL-C and VAI showed a lower validity. It is noteworthy that these results emphasize the possible influence of sex and age on the accuracy of evaluated indicators.

LAP, calculated using WC and fasting TG levels, was a robust tool to reflect central obesity and excessive lipid accumulation. Furthermore, accumulating evidence suggested that LAP could be used for the assessment of insulin resistance, and was closely associated with the development of DM, hypertension and metabolic syndrome (MS) [30,31]. In this case, given the higher AUC and YI, LAP presented relatively high identification ability for IR, and the cut-off value for men was 48.70 and 29.25 for women. Studies from different populations have reported the similar predictive ability of LAP for IR. A study from Italy showed that LAP was a more reliable discriminator of insulin resistance in clinical settings (AUC: 0.728 [0.692–0.762]), and had a stronger correlation with insulin compared with TyG, TG/HDL-C and VAI [32], which is in accord with our findings. In addition, a study conducted in Japan found that these tested variables had stronger association with IR in women than in men [33]. There is difference for the association between insulin resistance and body fat distribution in different ethnic groups, and in different age, sex population [34]. Further prospective investigations are needed to explain this concern.

TyG, exploited by Simental-Mendia *et al.* [14], is a simple, available surrogate for identifying IR, and showed an association with diabetes, hypertension, non-alcoholic fatty liver disease and atherosclerosis [35–38]. Previous study have showed that TyG is a superior tool for discriminating individuals with IR than VAI and LAP, and presented the greatest value of AUC (0.709 in men, and 0.711 in women) [16]. However, in the present study, no significant difference was found compared with LAP. One of the explanations may be the key mechanism of insulin resistance consisting of glucotoxicity and lipotoxicities [39,40]. Similar results were observed based on a Japanese population, which provide the cut-off value of TyG for men was 8.49 (AUC: 0.777 [0.746–0.807]) and 8.12 (AUC: 0.790 [0.737–0.843]) for women, respectively. Moreover, a stronger association with insulin resistance was observed in women than man [33], which is consistent with our study. Separate longitudinal studies in men and women are necessarily carried out on the capability of detecting IR for LAP and TyG in future studies. In this case, the cut-off value of TyG for men was 8.87, and the one for women was 8.50.

In comparison with other traditional lipid parameters, it has been reported that the sensitivity and specificity of TG/HDL-C to identify IR were almost the same as that of fasting insulin concentration [7]. It also seemed to be a better diagnostic tool in correlation with prevalent insulin resistance due to the key metabolic abnormalities consisting of hypertriglyceridaemia and low HDL-C in patients with IR [41,42]. In addition, several studies have suggested that TG/HDL-C could be used as a marker of cardio-metabolic disease and type 2 diabetes [8,43,44]. Nevertheless, when it comes to comparisons of other surrogates of predicting IR, possible influence of visceral fat distribution was not considered, which is reported to be closely correlated with risk of impaired glucose metabolism and diabetes. As a novel indicator to both assess visceral fat distribution and adipose tissue dysfunction, VAI was positively correlated with insulin resistance and β cell function in non-diabetic individuals [45–47]. Despite the positive correlation between TG/HDL-C, VAI and insulin resistance was observed in this study, significant difference compared with LAP and TyG was shown. Difference among them may be explained as follows: First, the correlation between TG/HDL-C and IR may differ according to ethnic origin, which indicate the gap in the applicability of the index in different groups [48]; second, there could be a greater error for those parameters, which are calculated based on anthropometric data. And finally, BMI, WC, fat, and liver fat demonstrated varieties for different ethnicity [49,50].

It is important to estimate a valid cut-off value for the clinical use. However, data for the cut-off value of LAP and TyG index for identifying IR are limited. In a Japanese study, the cut-off value for men and women was 24.1, 15.2 for LAP, and 8.79, 8.12 for TyG, respectively [33], which is lower than ours. This inconsistency may be explained by the age distribution and the difference of biochemical indexes among different races. Notably, the AUCs of four evaluated variables for female subjects were much higher than that for male subjects in this study population, whereas a higher cut-off value was observed in men. Possible explanations for this inconsistency could be the hormonal changes in women, and relationship between body fat distribution and insulin resistance by race [51], but further investigations are needed to clarify this collection. Besides, we also found a obvious decline of the diagnostic performance of four markers of IR with ageing, which indicated that the younger the subjects is, the better the diagnostic ability could have. In this research, we probed into the possible effect of age on the diagnostic ability of IR, and corresponding cut-off values of each marker were provided for reference.

Our study had some limitations to be discussed. First, as a cross-sectional study with a relatively small sample size, potential cause and effect relationships could not be drawn. Second, Hyperinsulinemic-euglycemic clamp, the gold standard for evaluating insulin sensitivity, was not conducted in this study. In the present study, HOMA-IR was used as an alternative tool for identifying insulin resistance. Furthermore, the study population was only composed of Chinese people aged 40–60 years, and thus the results could not be generalized to other racial or ethnic population. Future prospective design is essential to confirm the impact of these findings on the characteristics of insulin resistance. And finally, the presence of anthropometric errors by unmeasured factors cannot be avoided.

## Conclusions

In conclusion, based on the findings of the present study, LAP and TyG have relatively high predictive ability in diagnosis of IR compared with VAI and TG/HDL-C, and no significant difference was observed between LAP and TyG in predicting insulin resistance. Given the fact that inexpensive measurements of waist circumference and easy-to-get TG in clinical practice, LAP and TyG could be used as simple and alternative methods to identify the individuals at risk for insulin resistance.

## Data Availability

The data that support the findings of this study are openly available in Zenodo at http://10.5281/zenodo.5105910.

## Abbreviations

TG/HDL-C: Triglyceride-to-HDL cholesterol ratio
LAP: lipid accumulation product
VAI: visceral adiposity index
TyG index: triglycerides/glucose index
IR: insulin resistance

## References

[1] Lebovitz HE. Insulin resistance: definition and consequences. Exp Clin Endocrinol Diabetes. 2001;109(Suppl 2):S135–S148. 11460565 10.1055/s-2001-18576

[2] DeFronzo RA, Ferrannini E. Insulin resistance. A multifaceted syndrome responsible for NIDDM, obesity, hypertension, dyslipidemia, and atherosclerotic cardiovascular disease. Diabetes Care. 1991;14(3):173–194. 2044434 10.2337/diacare.14.3.173

[3] Adnan E, Rahman IA, Faridin HP. Relationship between insulin resistance, metabolic syndrome components and serum uric acid. Diabetes Metab Syndr. 2019;13(3):2158–2162. 31235151 10.1016/j.dsx.2019.04.001

[4] Suf M, Z M, J A, et al. Relationship among obesity, blood lipids and insulin resistance in Bangladeshi adults. Diabetes Metab Syndr. 2019;13:444–449. 30641741 10.1016/j.dsx.2018.10.015

[5] DeFronzo RA, Tobin JD, Andres R. Glucose clamp technique: a method for quantifying insulin secretion and resistance. Am J Physiol. 1979;237(3):E214–E223. 382871 10.1152/ajpendo.1979.237.3.E214

[6] Wallace TM, Levy JC, Matthews DR. Use and abuse of HOMA modeling. Diabetes Care. 2004;27(6):1487–1495. 15161807 10.2337/diacare.27.6.1487

[7] McLaughlin T, Abbasi F, Cheal K, et al. Use of metabolic markers to identify overweight individuals who are insulin resistant. Ann Intern Med. 2003;139(10):802–809. 14623617 10.7326/0003-4819-139-10-200311180-00007

[8] McLaughlin T, Reaven G, Abbasi F, et al. Is there a simple way to identify insulin-resistant individuals at increased risk of cardiovascular disease? Am J Cardiol. 2005;96(3):399–404. 16054467 10.1016/j.amjcard.2005.03.085

[9] Bertsch RA, Merchant MA. Study of the use of lipid panels as a marker of insulin resistance to determine cardiovascular risk. The Permanente Journal. 2015;4:4–10. 10.7812/TPP/14-237 PMC4625988 26517432

[10] Li C, Ford ES, Meng Y-X, et al. Does the association of the triglyceride to high-density lipoprotein cholesterol ratio with fasting serum insulin differ by race/ethnicity? Cardiovasc Diabetol. 2008;7:4. 18307789 10.1186/1475-2840-7-4 PMC2292689

[11] Amato MC, Giordano C, Galia M. Visceral adiposity index: a reliable indicator of visceral fat function associated with cardiometabolic risk. Diabetes Care. 2010;33(4):920–922. 20067971 10.2337/dc09-1825 PMC2845052

[12] Pekgor S, Duran C, Berberoglu U, et al. The role of visceral adiposity index levels in predicting the presence of metabolic syndrome and insulin resistance in overweight and obese patients. Metab Syndr Relat Disord. 2019;17(5):296–302. 30932744 10.1089/met.2019.0005

[13] Koloverou E, Panagiotakos DB, Kyrou I. Visceral adiposity index outperforms common anthropometric indices in predicting 10-year diabetes risk: results from the ATTICA study. Diabetes Metab Res Rev. 2019;35(6):e3161. 30912290 10.1002/dmrr.3161

[14] Simental-Mendía LE, Rodríguez-Morán M, Guerrero-Romero F. The product of fasting glucose and triglycerides as surrogate for identifying insulin resistance in apparently healthy subjects. Metab Syndr Relat Disord. 2008;6(4):299–304. 19067533 10.1089/met.2008.0034

[15] Toro-Huamanchumo CJ, Urrunaga-Pastor D, Guarnizo-Poma M. Triglycerides and glucose index as an insulin resistance marker in a sample of healthy adults. Diabetes Metab Syndr. 2019;13(1):272–277. 30641711 10.1016/j.dsx.2018.09.010

[16] Du T, Yuan G, Zhang M, et al. Clinical usefulness of lipid ratios, visceral adiposity indicators, and the triglycerides and glucose index as risk markers of insulin resistance. Cardiovasc Diabetol. 2014;13:146. 25326814 10.1186/s12933-014-0146-3 PMC4209231

[17] Vasques ACJ, Novaes FS, de Oliveira MDS, et al. TyG index performs better than HOMA in a Brazilian population: a hyperglycemic clamp validated study. Diabetes Res Clin Pract. 2011;93(3):e98–e100. 21665314 10.1016/j.diabres.2011.05.030

[18] Kahn HS. The lipid accumulation product is better than BMI for identifying diabetes. Diabetes Care. 2006;29(1):151–153. 16373916 10.2337/diacare.29.1.151

[19] Mazidi M, Kengne A-P, Katsiki N, et al. Lipid accumulation product and triglycerides/glucose index are useful predictors of insulin resistance. J Diabetes Complicat. 2018;32(3):266–270. 10.1016/j.jdiacomp.2017.10.007 29395839

[20] Xia C, Li R, Zhang S, et al. Lipid accumulation product is a powerful index for recognizing insulin resistance in non-diabetic individuals. Eur J Clin Nutr. 2012;66(9):1035–1038. 22781025 10.1038/ejcn.2012.83

[21] Organisation WH. Guidelines for controlling and monitoring the tobacco epidemic. Geneva：Tobacco or Heahh Programme. 1997.

[22] Matthews DR, Hosker JP, Rudenski AS, et al. Homeostasis model assessment: insulin resistance and beta-cell function from fasting plasma glucose and insulin concentrations in man. Diabetologia. 1985;28(7):412–419. 3899825 10.1007/BF00280883

[23] Xing XY, Yang WY, Yang ZJ. The diagnostic significance of homeostasis model assessment of insulin resistance in metabolic syndrome among subjects with different glucose tolerance (Chinese). Chin J Diabetes. 2004;3:182–186.

[24] Ainsworth BE, Haskell WL, Whitt MC, et al. Compendium of physical activities: an update of activity codes and MET intensities. Med Sci Sports Exerc. 2000;32(9 Suppl):S498–S504. 10993420 10.1097/00005768-200009001-00009

[25] Alberti KG, Zimmet P, Shaw J. The metabolic syndrome—a new worldwide definition. The Lancet (British Edition). 2005;366(9491):1059–1062. 10.1016/S0140-6736(05)67402-8 16182882

[26] Yang GR, Yuan SY, Fu HJ. Neck circumference positively related with Central obesity, overweight, and metabolic syndrome in Chinese subjects with type 2 diabetes: Beijing community diabetes study 4. Diabetes Care. 2010;33(11):2465–2467. 20724650 10.2337/dc10-0798 PMC2963514

[27] Poulter NR, Prabhakaran D, Caulfield M. Hypertension. The Lancet. 2015;386(9995):801–812. 10.1016/S0140-6736(14)61468-9 25832858

[28] DeLong ER, DeLong DM, Clarke Pearson DL. Comparing the areas under two or more correlated receiver operating characteristic curves: a nonparametric approach. Biometrics. 1988;44(3):837–845. 3203132

[29] Ms P. The statistical evaluation of medical tests for classification and prediction. Technometrics. 2005;2:245–245.

[30] Nascimento-Ferreira MV, Rendo-Urteaga T, Vilanova-Campelo RC, et al. The lipid accumulation product is a powerful tool to predict metabolic syndrome in undiagnosed Brazilian adults. Clin Nutr. 2017;36(6):1693–1700. 28081980 10.1016/j.clnu.2016.12.020

[31] Bala C, Gheorghe-Fronea O, Pop D, et al. The association between six surrogate insulin resistance indexes and hypertension: a population-based study. Metab Syndr Relat Disord. 2019;17(6):328–333. 31034338 10.1089/met.2018.0122

[32] Fiorentino TV, Marini MA, Succurro E, et al. Relationships of surrogate indexes of insulin resistance with insulin sensitivity assessed by euglycemic hyperinsulinemic clamp and subclinical vascular damage. BMJ Open Diabetes Res Care. 2019;7(1):e000911. 10.1136/bmjdrc-2019-000911 PMC6861112 31798905

[33] Nakagomi A, Sunami Y, Kawasaki Y, et al. Sex difference in the association between surrogate markers of insulin resistance and arterial stiffness. J Diabetes Complicat. 2020;34(6):107442. 10.1016/j.jdiacomp.2019.107442 31668590

[34] Araneta MRG, Barrett Connor E. Ethnic differences in visceral adipose tissue and type 2 diabetes: Filipino, African-American, and white women. Obesity (Silver Spring, Md.). 2005;13:1458–1465. 10.1038/oby.2005.176 16129729

[35] Lee DY, Lee ES, Kim JH, et al. Predictive value of triglyceride glucose index for the risk of incident diabetes: a 4-Year retrospective longitudinal study. Plos One. 2016;11(9):e0163465. 27682598 10.1371/journal.pone.0163465 PMC5040250

[36] Zhang S, Du T, Zhang J, et al. The triglyceride and glucose index (TyG) is an effective biomarker to identify nonalcoholic fatty liver disease. Lipids Health Dis. 2017;16(1):15. 28103934 10.1186/s12944-017-0409-6 PMC5248473

[37] Lee SH, Kwon HS, Park YM, et al. Predicting the development of diabetes using the product of triglycerides and glucose: the chungju metabolic disease cohort (CMC) study. Plos One. 2014;9(2):e90430. 24587359 10.1371/journal.pone.0090430 PMC3938726

[38] Kim MK, Ahn CW, Kang S, et al. Relationship between the triglyceride glucose index and coronary artery calcification in Korean adults. Cardiovasc Diabetol. 2017;16(1):108. 28830471 10.1186/s12933-017-0589-4 PMC5568209

[39] Rossetti L, Giaccari A, DeFronzo RA. Glucose toxidity. Diabetes Care. 1990;13(6):610–630. 2192847 10.2337/diacare.13.6.610

[40] DeFronzo RA. Insulin resistance, lipotoxicity, type 2 diabetes and atherosclerosis: the missing links. The Claude Bernard lecture 2009. Diabetologia. 2010;53(7):1270–1287. 20361178 10.1007/s00125-010-1684-1 PMC2877338

[41] Rashid S, Watanabe T, Sakaue T, et al. Mechanisms of HDL lowering in insulin resistant, hypertriglyceridemic states: the combined effect of HDL triglyceride enrichment and elevated hepatic lipase activity. Clin Biochem. 2003;36(6):421–429. 12951168 10.1016/s0009-9120(03)00078-x

[42] Ma M, Liu H, Yu J, et al. Triglyceride is independently correlated with insulin resistance and islet beta cell function: a study in population with different glucose and lipid metabolism states. Lipids Health Dis. 2020;19(1):121. 32487177 10.1186/s12944-020-01303-w PMC7268278

[43] Borrayo G, Basurto L, González-Escudero E, et al. Tg/Hdl-C ratio as Cardio-Metabolic biomarker even in normal weight women. Acta Endocrinol (Buchar). 2018;14(2):261–267. 31149268 10.4183/aeb.2018.261 PMC6516523

[44] Zhang Y, Qin P, Lou Y, et al. Association of TG/HDLC ratio trajectory and risk of type 2 diabetes: a retrospective cohort study in China. J Diabetes. 2020;13(5):402–412. 10.1111/1753-0407.13123 33074586

[45] Al-Daghri NM, Al-Attas OS, Alokail MS, et al. Visceral adiposity index is highly associated with adiponectin values and glycaemic disturbances. Eur J Clin Invest. 2013;43(2):183–189. 23278387 10.1111/eci.12030

[46] Chen C, Xu Y, Guo ZR, et al. The application of visceral adiposity index in identifying type 2 diabetes risks based on a prospective cohort in China. Lipids Health Dis. 2014;13(1):108. 25002013 10.1186/1476-511X-13-108 PMC4108974

[47] Sung HH, Park CE, Gi MY, et al. The association of the visceral adiposity index with insulin resistance and beta-cell function in Korean adults with and without type 2 diabetes mellitus. Endocr J. 2020;67(6):613–621. 32161204 10.1507/endocrj.EJ19-0517

[48] Kim-Dorner SJ, Deuster PA, Zeno SA, et al. Should triglycerides and the triglycerides to high-density lipoprotein cholesterol ratio be used as surrogates for insulin resistance? Metabolism. 2010;59(2):299–304. 19796777 10.1016/j.metabol.2009.07.027

[49] Heymsfield S, Peterson C, Thomas D, et al. Why are there race/ethnic differences in adult body mass index-adiposity relationships? A quantitative critical review. Obes Rev. 2016;17(3):262–275. 26663309 10.1111/obr.12358 PMC4968570

[50] Wulan S, Westerterp K, Plasqui G. Ethnic differences in body composition and the associated metabolic profile: a comparative study between Asians and Caucasians. Maturitas. 2010;65(4):315–319. 20079586 10.1016/j.maturitas.2009.12.012

[51] Geer E, Shen W. Gender differences in insulin resistance, body composition, and energy balance. Gend Med. 2009;6(Suppl 1):60–75. 19318219 10.1016/j.genm.2009.02.002 PMC2908522

